# Maternally Perceived Fetal Movement and Computerized Cardiotocography Parameters: What’s the Link? A Multicenter Pilot Study on Fetal Health

**DOI:** 10.3390/diagnostics16193081

**Published:** 2026-09-23

**Authors:** Marco La Verde, Marco Torella, Davide Pisani, Lorenza Driul, Mario Fordellone, Petra Hanulíková, Lucia Pasquini, Liliana Mariani, Mattia Dominoni, Barbara Gardella

**Affiliations:** 1 Department of Woman, Child and General and Specialized Surgery, University of Campania “Luigi Vanvitelli”, 80138 Naples, Italy; marco.torella@unicampania.it (M.T.); davidepisani94@gmail.com (D.P.); 2 Clinic of Obstetrics and Gynecology, “Santa Maria della Misericordia” University Hospital, Azienda Sanitaria Universitaria Friuli Centrale, 33100 Udine, Italy; lorenza.driul@uniud.it; 3 Department of Medicine, University of Udine, 33100 Udine, Italy; 4 Medical Statistics Unit, University of Campania “Luigi Vanvitelli”, 80138 Naples, Italy; mario.fordellone@unicampania.it; 5 Institute for the Care of Mother and Child, Third Faculty of Medicine, Charles University, 147 00 Prague, Czech Republic; hanulikovap@gmail.com; 6 Fetal Medicine Unit, Department for Woman and Child Health, Careggi University Hospital, 50134 Florence, Italy; pasquinil@aou-careggi.toscana.it; 7 Department of Clinical, Surgical, Diagnostic and Paediatric Sciences, University of Pavia, 27100 Pavia, Italy; liliana.mariani01@universitadipavia.it (L.M.); matti.domino@gmail.com (M.D.); barbara.gardella@gmail.com (B.G.); 8 Department of Obstetrics and Gynecology, Fondazione IRCCS Policlinico San Matteo, 27100 Pavia, Italy

**Keywords:** pregnancy, fetal movement, computerized cardiotocography, cardiotocography, maternal perception, active fetal movements, fetal health monitoring, prenatal care

## Abstract

**Background**: Maternal perception of fetal movement (FM) is an indirect indicator of fetal well-being. The aim was to investigate the relationship between computerized cardiotocography (cCTG) parameters and maternal perception of FM. **Methods**: We conducted a prospective, multicentric observational study at two tertiary referral hospitals. After cCTG, all women completed a questionnaire assessing maternal perception of FM and fetal hiccups. The primary outcome was the relation between multiple cCTG parameters and maternal perceptions of FM. **Results**: We included 419 patients. Women who reported increased FM frequency perception during the previous two weeks had an increased number of accelerations (*p* = 0.0099). The Dawes–Redman criteria need more time to be satisfied in pregnant women with no hiccup perception (*p* = 0.0063). These associations were confirmed after adjustment for pre-pregnancy BMI, gestational diabetes, and gestational hypertension. The association between two episodes of reduced FM after 26 weeks and high long-term variability (*p* = 0.032) was not confirmed after adjustment. No other statistical correlation was observed. **Conclusions**: In this pilot multicenter study, fetal well-being assessed with cCTG was associated with increased perceived FM and perception of fetal hiccups. In future phases, this research will integrate maternal FM perception, cCTG, prenatal ultrasound, and neonatal outcomes to provide a comprehensive evaluation of the maternal perception of FM.

## 1. Introduction

Prenatal care based on the patient’s history, physical examination, ultrasound, laboratory analysis, and cardiotocography (CTG) represent critical components of monitoring fetal well-being [1,2]. CTG records the fetal heart rate (FHR) and uterine contractions [3]. FHR variability is a marker of fetal well-being, reflecting the sympathetic and parasympathetic nervous systems balance [4]. Based on FHR, CTG identifies fetal hypoxemia and supports obstetric management [5,6]. The principal CTG limitation is subjective interpretation, with substantial intra- and inter-observer variability [7]. Computerized CTG (cCTG) was introduced to perform an objective FHR analysis [8]. cCTG analyzes FHR with advanced algorithms, providing a quantitative assessment of FHR parameters [9]. cCTG enables quantitative assessment of short-term variability (STV), supporting fetal growth restriction (FGR) management and high-risk pregnancy [10]. The systematic fetal evaluation through cCTG parameters includes the Dawes–Redman criteria, which support the fetal well-being assessment [11,12]. This approach provides a more standardized and objective CTG interpretation [13]. Whether computerized methods improve perinatal outcomes remains uncertain [14,15]. Maternal perception of fetal movements (FM) is a non-invasive, intuitive and cost-effective indicator of fetal well-being, reflecting the integrity of the central nervous system [16]. Decreased FM may precede serious fetal complications [17]. Maternal perception of FM has long represented one of the oldest modalities for prenatal non-invasive fetal well-being monitoring. Decreased fetal activity has been reported to precede adverse pregnancy outcomes [18]. Assessment based on maternal perception of FM is subjective [19]. On the other hand, cCTG provides a more standardized tool for fetal monitoring [20]. Using advanced algorithms to analyze FHR patterns, cCTG has shown promise in reducing inter- and intra-observer variability associated with traditional visual interpretation of CTG [21]. The relationship between maternal perception of FM and cCTG parameters remains poorly investigated. This lacuna needs to be filled, as the integration of these approaches may offer a more complete understanding of fetal well-being [22]. We aimed to address this gap by integrating subjective (maternal perception of FM) and objective monitoring techniques (cCTG) into a combined cohesive evidence-based practice. We examined for the first time the relation between cCTG parameters and maternal FM perception to improve prenatal care and to better understand the real impact of maternal perception on cCTG parameters. This pilot study provides the basis for further research, in which maternal perception of FM associated with the cCTG parameters will be compared with other methods of FM evaluation. In future research, we will integrate maternal FM perception and cCTG parameters with the neonatal outcomes (Apgar scores or NICU admission) and the ultrasound exams (through amniotic fluid, fetal Doppler velocimetry, and fetal biophysical profile score evaluation). All these analyses aim to improve interpretation of the maternal FM perception.

## 2. Materials and Methods

This prospective multicentric observational study was conducted at the Gynecology and Obstetrics Unit of the University of Campania “Luigi Vanvitelli” of Naples and at the University Study of Pavia. The protocol design, collection, analysis, and interpretation of data, as well as drafting, and subsequent revisions followed the Strengthening the Reporting of Observational Studies in Epidemiology (STROBE) Statement guidelines for reporting observational studies. All pregnancies, between 1 August 2024 and 20 August 2025, admitted to our institution’s cardiotocography ambulatory were invited to participate.

We excluded the following obstetric and fetal conditions: preterm gestation or premature rupture of membranes, multiple pregnancies and fetuses with malformations. Those conditions were excluded to reduce their influence on the maternal perception of FM or on cardiotocography parameters. Upon their first access to the cardiotocography ambulatory, the participants were registered and given written informed consent. Socio-demographic characteristics (gestational age, weight gain, pre-gestational BMI, age, smoker, maternal ethnic group) and obstetric data (gravidity, parity, previous cesarean section, induced abortion or miscarriage, diseases observed during the pregnancy, and fetal gender) were collected for each enrolled pregnant participant. Maternal perception of fetal movements (frequency and strength of FM) was assessed through a physician-administered questionnaire (D.P. or L.M.) (Table 1). The physician-administered questionnaire was distinct from the FM registered during the CTG. The questionnaire explored the changes in strength and frequency in the last 2 weeks of the maternal FM perception categorized as increased, decreased, or remained the same (Table 1). Unusual baby’s movements from 26 weeks and vigorous movement in the last two weeks also were explored (Table S1). All questions assessing maternal perception of fetal movements were adopted in accordance with previous studies, including Heazell et al., who validated these questions [23]. The Sonicaid Team3 system was the cCTG adopted (Huntleigh Healthcare Ltd., Cardiff, UK). Parameters evaluated in computerized CTG: Fetal movement per hour, accelerations, decelerations, high and low episodes, LTV, and STV were registered for each pregnancy. The primary outcome was the relationship between integrate multiple parameters of computerized CTG and the different maternal perceptions of FM. Continuous variables were reported as either means and standard deviation or median and interquartile ranges (IQRs) according to their distribution, as assessed by the Shapiro–Wilk normality test. Categorical variables were reported as absolute frequencies and percentages. For more than two groups, one-way ANOVA was used for comparisons of normally distributed variables, while the Kruskal–Wallis test was chosen for non-normally distributed variables. Multivariable models were applied for cCTG outcomes with significant univariate associations, including the corresponding maternal FM perception variable as the primary predictor and adjusting for pre-pregnancy BMI, gestational diabetes, and gestational hypertension (Table S2). Linear regression was used for LTV, negative binomial regression for the number of accelerations, and Gamma regression with a log link for the time required to satisfy the Dawes–Redman criteria, given its right-skewed distribution (Table S2). Adjusted estimates with 95% CI were reported, together with overall *p*-values for the perception variables obtained from likelihood-ratio or F tests. All the statistical values equal to or smaller than 0.05 were considered statistically significant. The analysis was conducted using R statistical software (version 4.1.3; 10 March 2022). This study was carried out with full respect for international standards of good clinical practice regarding the Helsinki Declaration and national and local regulations. This study was registered on ClinicalTrial.gov (identifier: NCT04397874). Appropriate approval was taken from the Ethics Committee of the University of Campania “Luigi Vanvitelli” (protocol number 0020184/i; date: 23 July 2024).

## 3. Results

A total of 482 women were identified as eligible participants. Sixty-three women were excluded based on the exclusion criteria (Figure 1). The demographic characteristics of the study population have been presented in detail in Table 1. The mean gestational age at the cardiotocography was 38.2 weeks (SD 1.0) in a population that was mainly term. The mean gestational weight gain was 10.4 kg (SD 6.1), and the mean body mass index (BMI) before pregnancy was 25.6 kg/m^2^ (SD 6.1). Of these participants, the majority were Caucasian at 83%, followed by South Asians at 8.4%, Black at 4.1%, Afro-Caribbeans at 3.3%, and East Asians at 1.4%. The mean maternal age was 32.1 (SD 5.5) years. The obstetric history revealed that 40% were primigravida (Table 1).

A previous cesarean section was reported in 16% of the study population. A history of one or more miscarriages was reported in 23% of the study population. A history of abortion was reported in 9% of the study population. The fetal sex was 52% male and 48% female. A total of 13% of pregnant women reported smoking during pregnancy. A total of 33.8% of the pregnant women were affected by gestational diabetes, and 11.2% had gestational hypertension or fetal growth restriction (Table 1).

Figure 2’s correlation matrix illustrates correlations between principal computerized cardiotocography parameters. Positive correlations were observed between cCTG duration and High Episodes (0.40, *p*-value 0.0001) and between LTV and STV (0.86, *p*-value 0.0001); one variable increasing coincides with increases in the other variable. Negative correlations were found in cCTG duration and STV (−0.26, *p*-value 0.001) or between Low Episodes and LTV (−0.58, *p*-value 0.0001), showing an inverse correlation.

Figure 3 shows the boxplots of eight parameters (Accelerations, Dawes–Redman, FHR baseline, High Episodes, Low Episodes, LTV, MAF per hour, and STV) stratified on the responses to “Was there any time from 26 weeks of pregnancy that your baby’s movements were less than usual?” (No, One time, Two times, Three or more times). All the parameters had no statistically significant differences, except the LTV (*p* = 0.032) between the four categories of responses, suggesting a correlation between LTV levels and women’s perceptions of decreased fetal movements (Figure 3). Women who reported less movement from 26 weeks of pregnancy than the usual “Two times” displayed higher LTV values than those who reported one reduction (Figure 4).

Considering the last 2 weeks’ frequency of the baby’s movements, categorized as Equal, Decreased, or Increased, the boxplots of eight parameters, Accelerations, Dawes–Redman, FHR baseline, High Episodes, Low Episodes, LTV, MAF per hour, and STV, showed significant differences across these three groups (Figure 5). Accelerations show a statistically significant difference (*p* = 0.0099) and reflect that increased fetal movement frequency is related to an increased number of accelerations. In contrast, Dawes–Redman (*p* = 0.82), FHR baseline (*p* = 0.78), High Episodes (*p* = 0.19), Low Episodes (*p* = 0.30), LTV (*p* = 0.69), and MAF per hour (*p* = 0.57) showed no statistical differences between the groups (Figure 5).

The boxplot of accelerations, stratified by the perceived frequency of the baby’s movements in the last 2 weeks, showed more accelerations in the group of mothers with an increased baby’s movement perception (Figure 6).

Across all cCTG parameters (Accelerations: *p* = 0.14, Dawes–Redman: *p* = 0.65, FHR baseline: *p* = 0.58, High Episodes: *p* = 0.76, Low Episodes: *p* = 0.22, LTV: *p* = 0.10, MAF per hour: *p* = 0.33, STV: *p* = 0.38), none showed statistical significance (*p* < 0.05) (Figure S1).

These findings suggest no association between the perceived strength of fetal movements and changes in these cCTG parameters (Figure S1).

Considering the fetal hiccup perception in the last 2 weeks (Figure S2), the Dawes–Redman criteria were satisfied less rapidly in the group of patients with no hiccup perception (*p* = 0.0063), suggesting that no perceived hiccup is associated with more time to reach the Dawes–Redman criteria with respect to pregnant women with hiccups (Figure 7). The multivariable analyses were adjusted for pre-pregnancy BMI, gestational diabetes, and gestational hypertension and confirmed the associations between perceived FM frequency with accelerations and the fetal hiccup perception with the Dawes–Redman criteria time (Table S2). While the univariate association between LTV and reduced FM from 26 weeks was not significant (overall *p* = 0.10, Table S2), gestational diabetes was independently associated with less time necessary to satisfy the Dawes–Redman criteria (adjusted time ratio 0.81, 95% CI 0.71–0.91, *p* < 0.001, Table S2).

## 4. Discussion

This study explores for the first time the relationship between maternally perceived changes in FM and cCTG parameters. Significant correlations were observed between cCTG parameters, like the Dawes–Redman criteria and accelerations, and maternal-reported changes in FM. These findings suggest that cCTG parameters may objectively quantify subjective maternal observations, bridging qualitative and quantitative assessments of fetal well-being. Our findings indicate a correlation between maternal perception of reduced fetal movement from 26 weeks of gestation and long-term variability (LTV). However, after adjustment for pre-pregnancy BMI, gestational diabetes, and gestational hypertension, this association was attenuated and no longer statistically significant. Women reporting two episodes of reduced FM after 26 weeks had higher LTV than pregnant women with a single episode of reduced FM. This isolated finding should be considered cautiously and should be explored in future research to understand if it represents a specific compensatory fetal response or not [24]. Previous studies have described fetal adaptive responses to hypoxemia and acid-base disturbances [25,26]. Our study revealed a correlation between the increased frequency of maternal perception of FM during the last 2 weeks and a higher number of accelerations. Accelerations are an indicator of fetal well-being, reflecting intact autonomic control. The link between the increased frequency and higher number of accelerations suggests that maternal perception is a good indirect sign of adequate physiological fetal status. Finally, our research observed a significant correlation between fetal hiccups and time to obatain the Dawes–Redman criteria. The Dawes–Redman criteria provide a computerized assessment of fetal well-being based on cCTG. Women who did not perceive fetal hiccups had a longer time to satisfy the Dawes–Redman criteria. These findings suggest that maternal perception of fetal hiccups may be associated with fetal neurobehavioral and autonomic status [27]. Maternal perception of FM has long been considered as an important indicator of fetal well-being [28]. Heazell et al. reported an association between changes in FM perception and adverse perinatal outcomes, including late stillbirth [29]. Piron-Dumitrascu et al. evidenced the role of FM monitoring combined with CTG, in high-risk pregnancy surveillance [22]. Our findings for the first time highlight the link between maternal fetal perception and cCTG parameters, thereby enabling a possible multidimensional approach to fetal well-being assessment [30]. This dual approach offers a more nuanced status of fetal well-being, particularly when traditional CTG parameters such as STV and episodes of high variability show a moderate correlation with FM perception [31]. Previous studies have outlined the inadequacy of the visual analysis of CTG [32], particularly the inter- and intra-observer variability, such as Imane et al., who explored algorithms and machine learning improvements, which support the cCTG sensibility to the fetal hypoxia detection [14]. Future studies are needed to explore the relationship between maternal perception of FM, cCTG and the delivery modality [33]. In addition technological advancements, such as artificial intelligence and machine learning algorithms, could improve our sensibility analysis [34,35]. Different studies have explored AI sensibility in CTG data evaluation [36,37]. Considering the literature, few studies have evaluated the predictive value of combined FM perception plus cCTG [38]. Saglam et al. showed that reduced FM may reflect underlying conditions that may develop later in pregnancy [39]. Maternal FM perception clinical utility remains ambiguous. First, maternal FM perception is subjective and may be influenced by other sensations and several factors such as BMI, smoking habits, and placental position, potentially influencing maternal perception [40,41]. Second, signal loss at higher BMI and subjectivity in CTG interpretation add additional limitations [42]. Das et al. have pointed out that clinical data on the association between decreased FM and perinatal outcomes remain limited, making comparisons among different studies difficult [2]. Our original article presented several strengths: no previous study has associated maternal FM perception with the cCTG evaluation; the multicentric prospective design of this study, with its different populations and clinical settings, gives generalizability to the study results and supports the integration of maternal FM perception across clinical settings. Thirdly, the population included is heterogeneous, reflecting real-word antenatal practice, and is adequate to support our conclusions. Lastly, the cCTG parameters offer an objective evaluation and analysis of the fetal status, reducing the intra and inter-observer variability, and a physician administered the questionnaire. To conclude, this research supports a multidimensional approach to fetal monitoring using technology and FM during pregnancy. The promotion of such integration may facilitate earlier recognition of fetal compromise and more individualized antenatal care. This is a multicenter pilot study. These preliminary data concerned the maternal perception of fetal movements with the cCTG parameters. Further stages in this project will use numerous quantitative methods, like ultrasound evaluation for amniotic fluid, fetal Doppler and fetal growth parameters [43,44]. This comprehensive approach will allow maternal perception of FM to be compared with more objective endpoints and outcome-based thresholds.

## 5. Conclusions

Increased perceived FM frequency during the previous two weeks and maternal perception of fetal hiccups were associated with objective cCTG measures of fetal well-being. These findings may support clinicians during prenatal care.

## Figures and Tables

**Figure 1:**
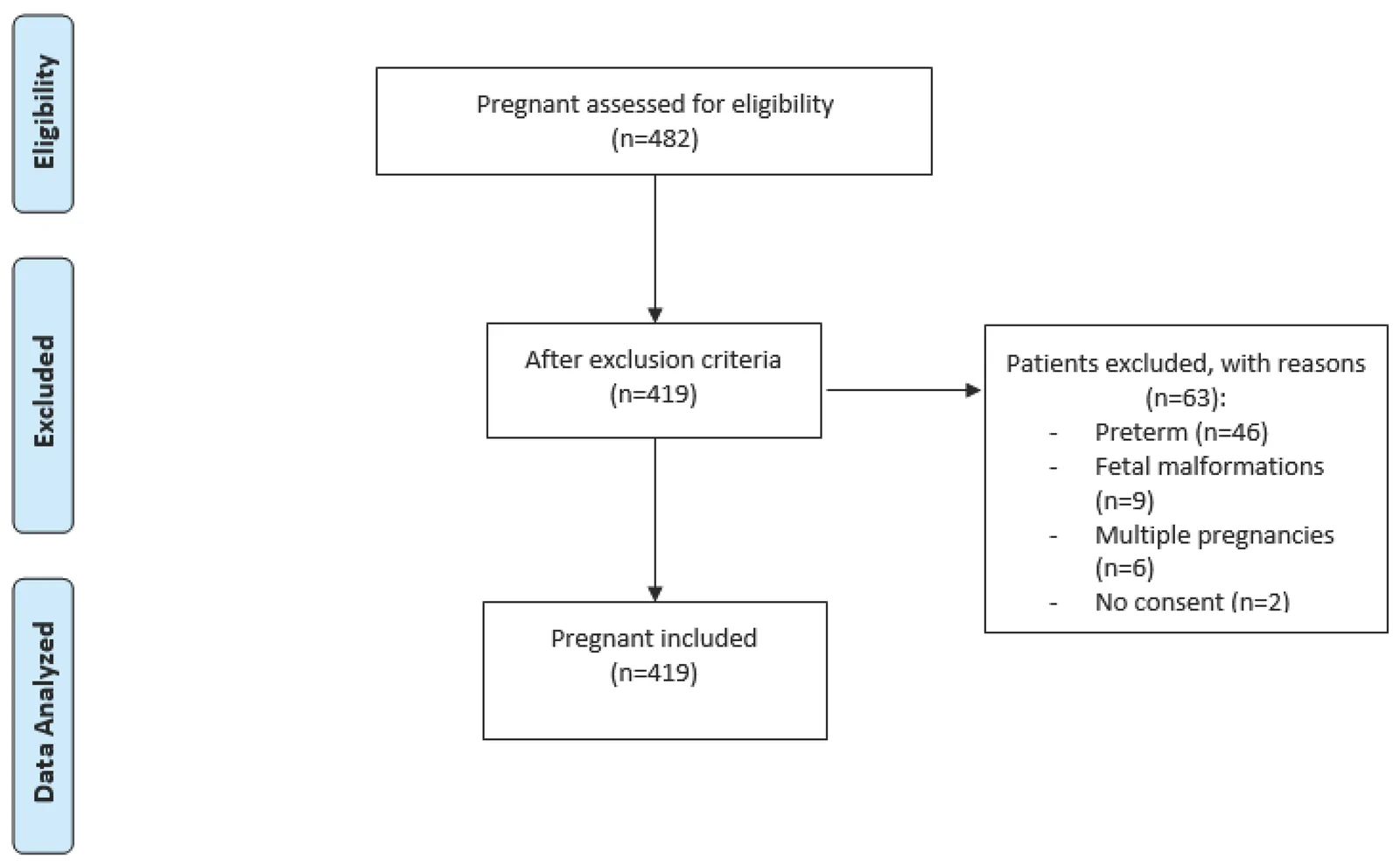
Study flowchart.

**Figure 2:**
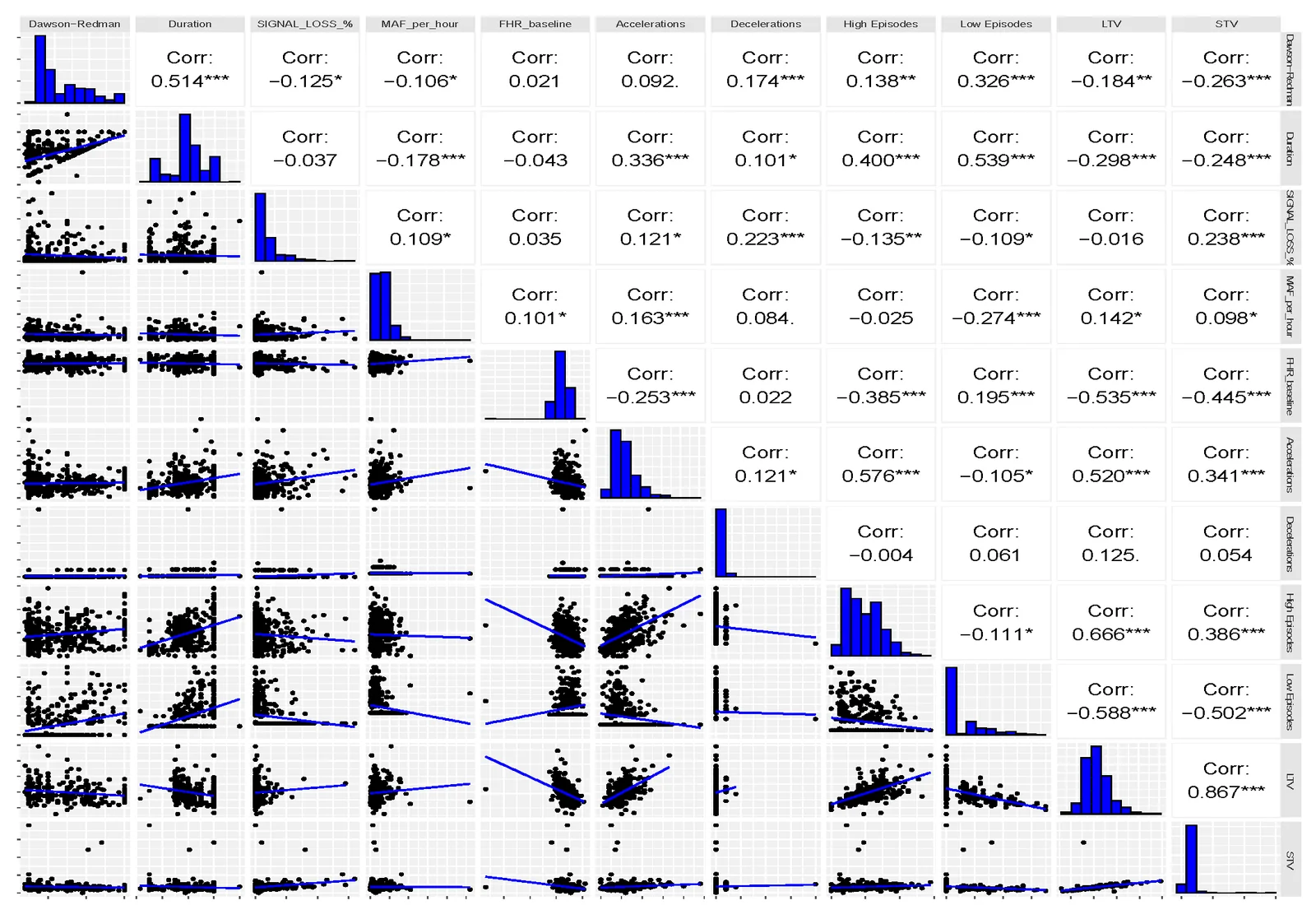
Pairs plot of multiple computerized cardiotocography (cCTG) parameters. Significance levels = . *p* < 0.1; * *p* < 0.05; ** *p* < 0.01; *** *p* < 0.001.

**Figure 3:**
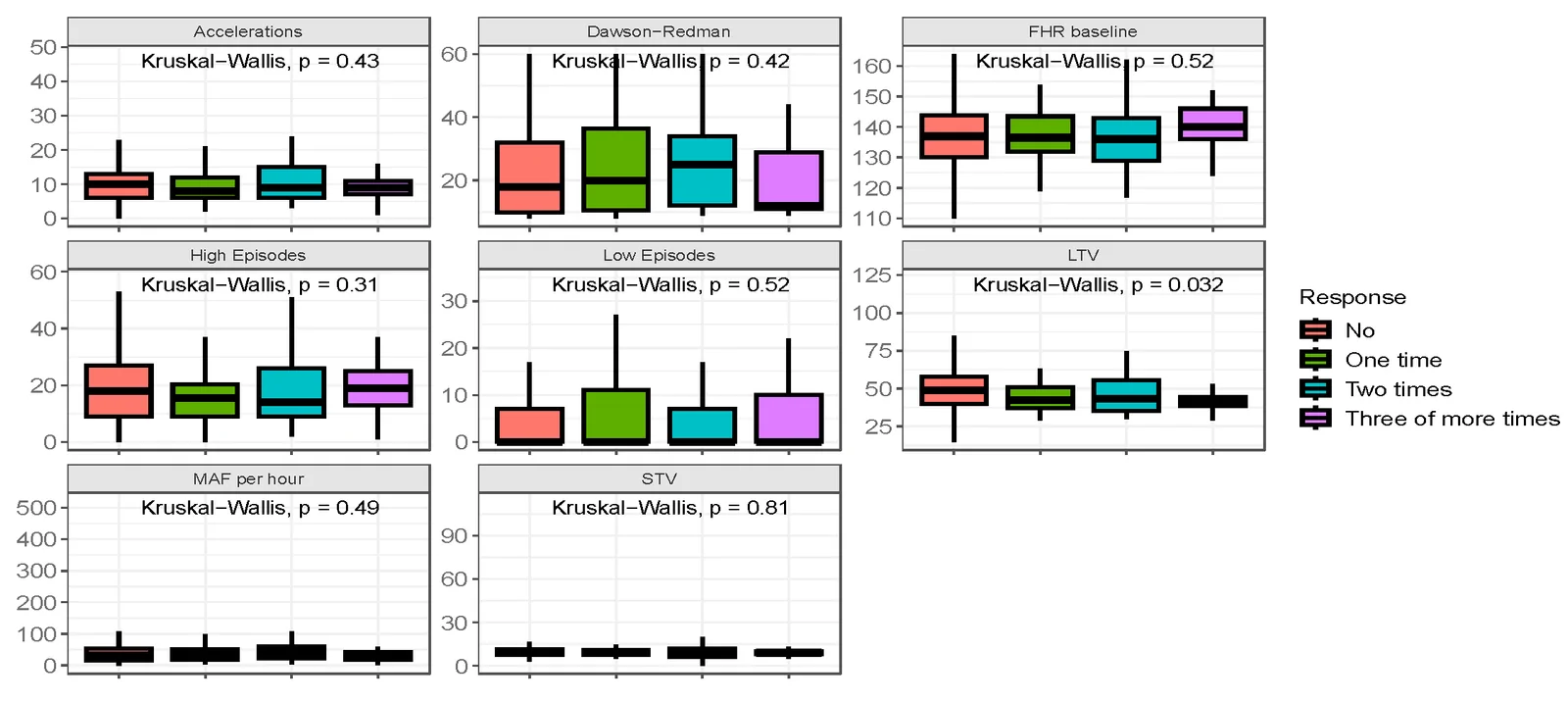
Boxplots of computerized cardiotocography parameters stratified pregnant participants’ responses to the question “Was there any time from 26 weeks of pregnancy that your baby’s movements were less than usual?”.

**Figure 4:**
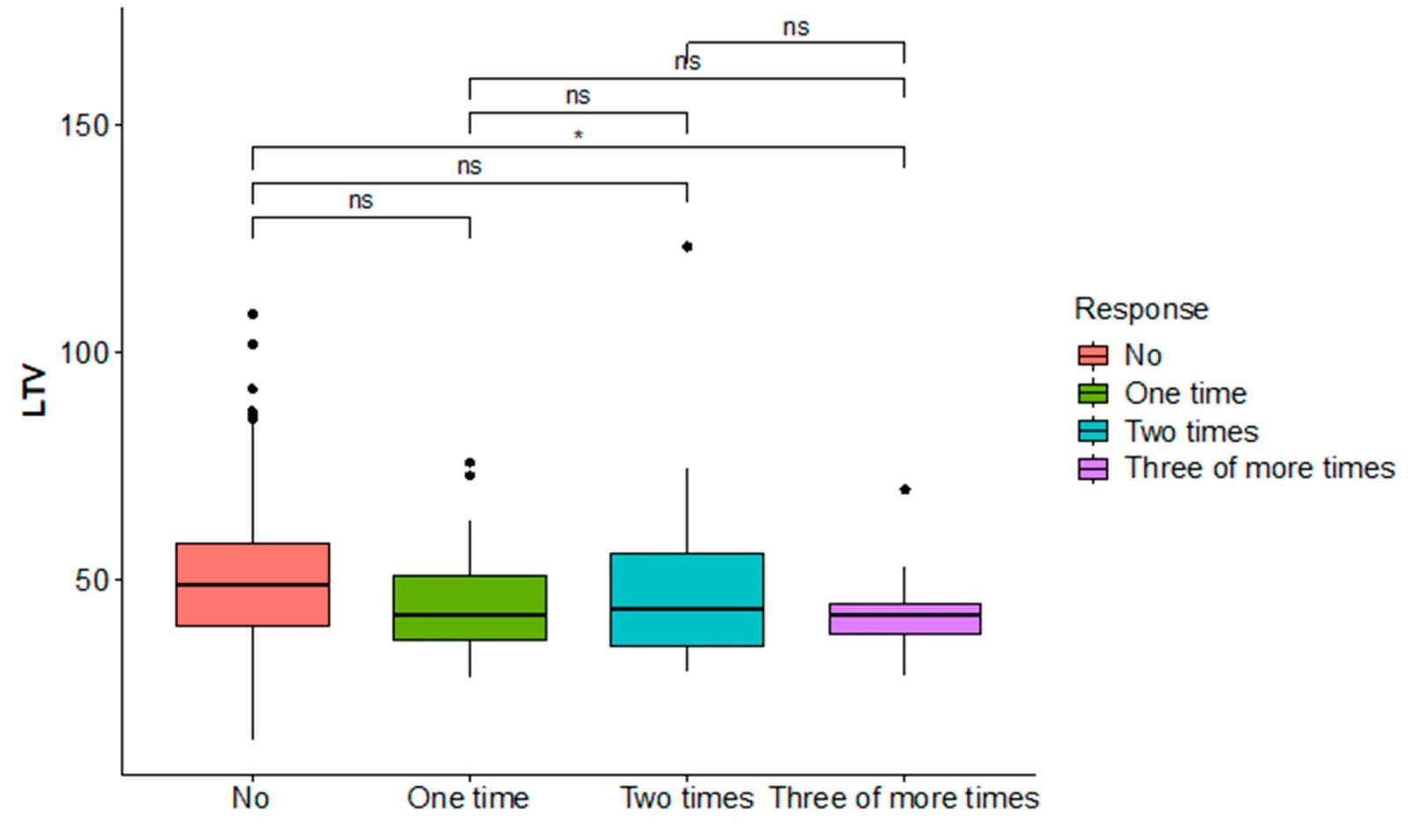
Boxplot of LTV values across four groups of participants who responded “No,” “One time,” “Two times,” or “Three or more times” to the question, “Was there any time from 26 weeks of pregnancy that your baby’s movements were less than usual?” ns, not significant (*p* ≥ 0.05); * *p* < 0.05.

**Figure 5:**
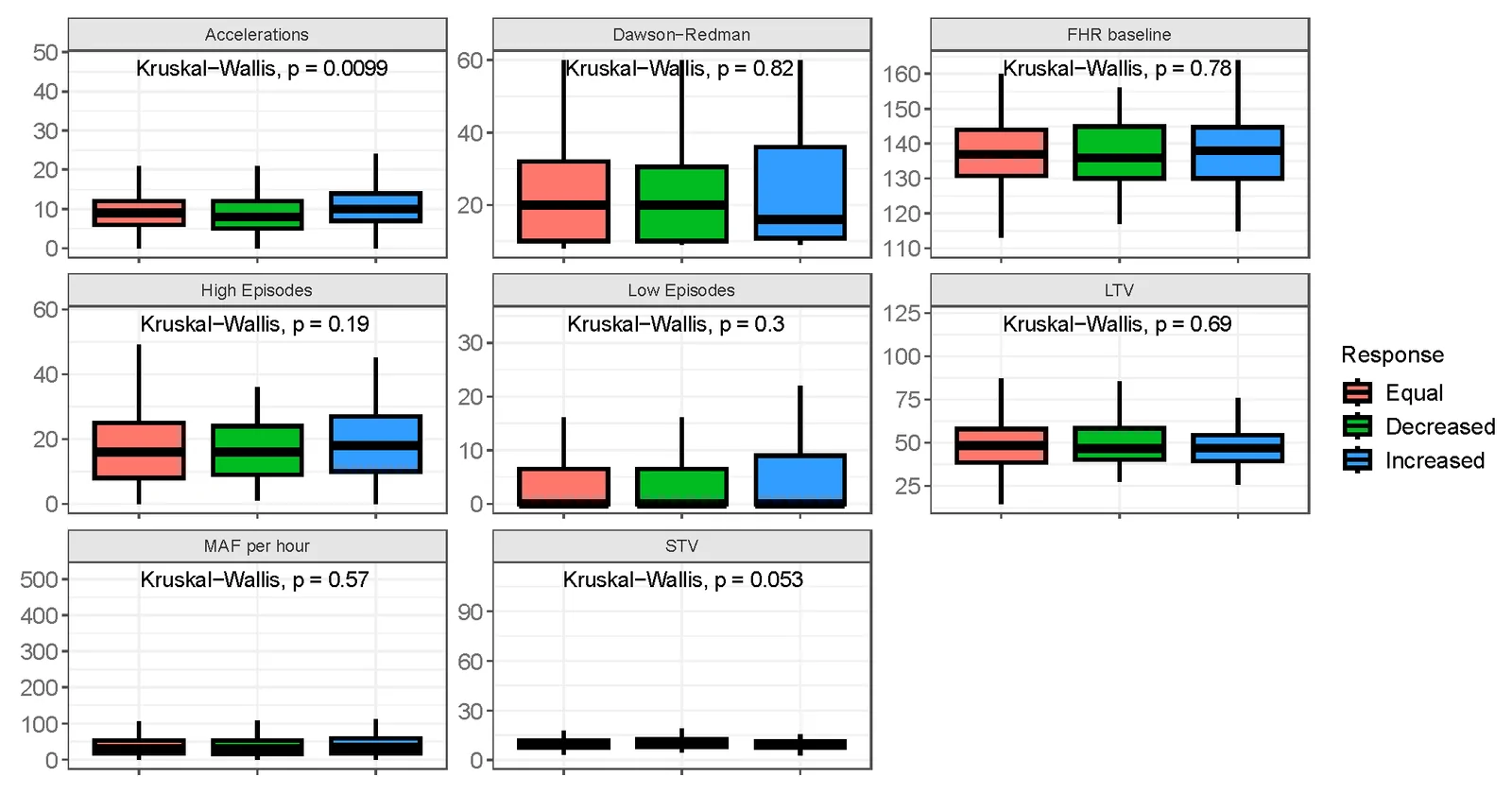
Boxplots of computerized cardiotocography parameters stratified pregnant participants’ responses to the question “In the last 2 weeks, did the frequency of your baby’s movements…? (categorized as Equal, Decreased, or Increased)”.

**Figure 6:**
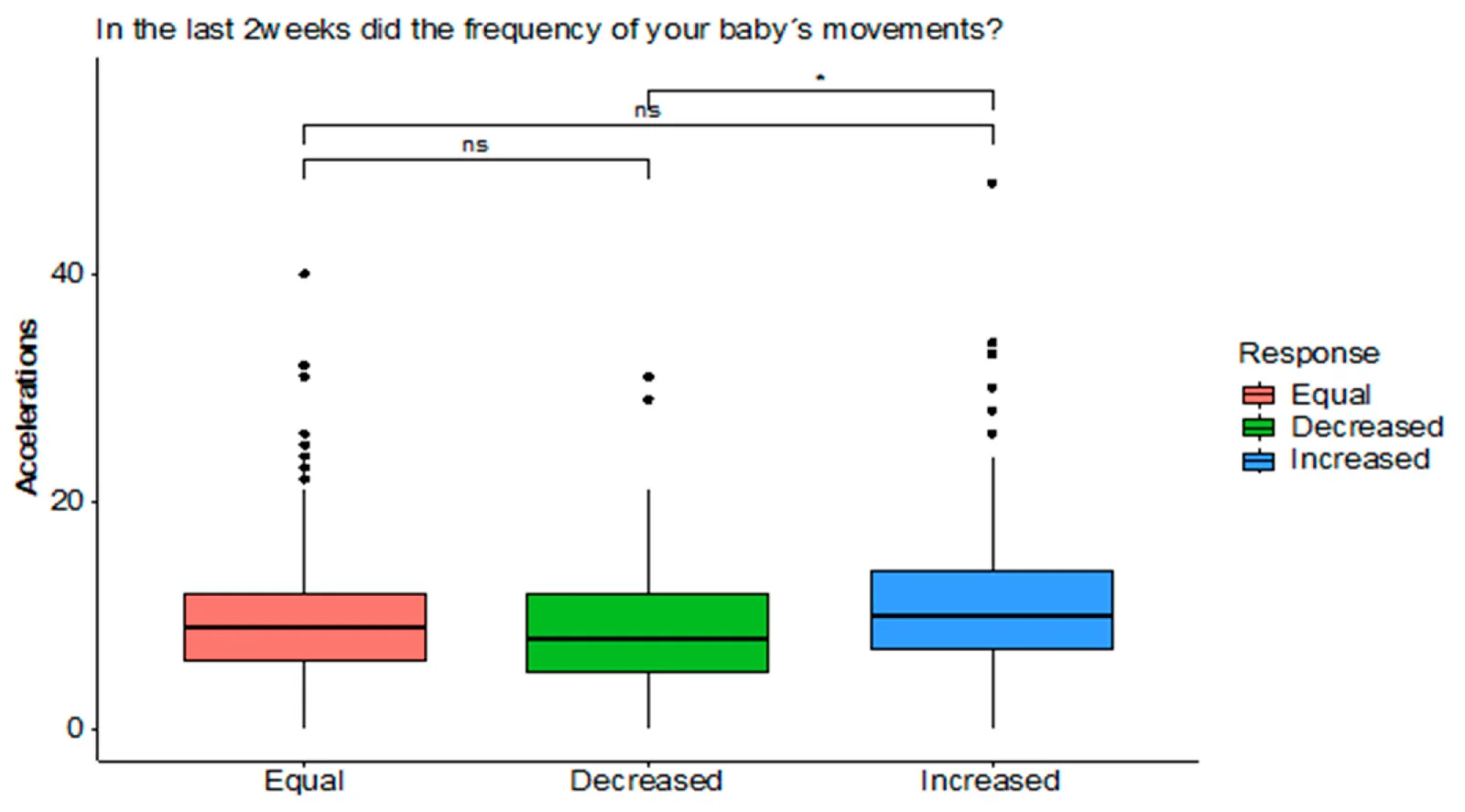
Boxplot of accelerations across three groups Equal, Decreased, or Increased of participants who responded to the question, “In the last 2 weeks, did the frequency of your baby’s movements…?” ns, not significant (*p* ≥ 0.05); * *p* < 0.05.

**Figure 7:**
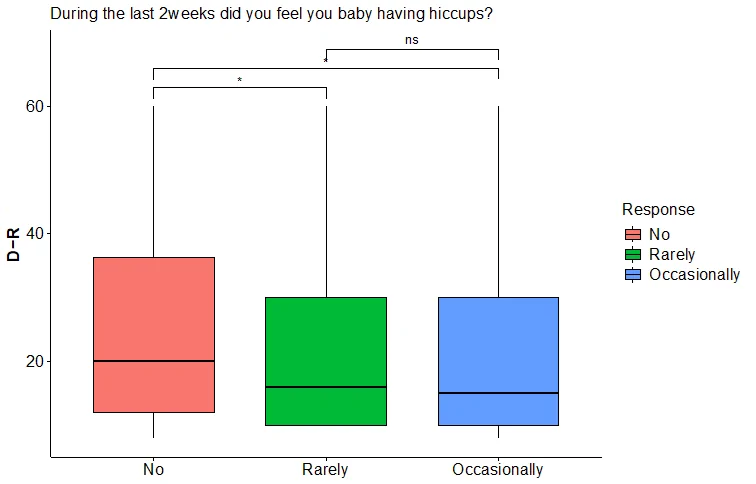
Boxplot of of the time required to obtain the Dawes–Redman criteria across three groups, No, Rarely, or Occasionally Hiccups perception. ns, not significant (*p* ≥ 0.05); * *p* < 0.05.

**Table 1:** Baseline characteristics of patients included.

Characteristic	*N* = 419 ^1^
Gestational age, Weeks	38.2 (1.0)
Gestational weight gain, Kg	10.4 (6.1)
Pre-pregnancy BMI, Kg/m^2^	25.6 (6.1)
Ethnicity	
Caucasian	347 (83%)
Black	17 (4.1%)
Afro-Caribbean	14 (3.3%)
South Asian	35 (8.4%)
East Asian	6 (1.4%)
Age, Years	32.1 (5.5)
Gravidity	
1	167 (40%)
2	133 (32%)
≥3	119 (28%)
Parity	
0	283 (68%)
1	94 (22%)
≥2	42 (10%)
Previous cesarean section	
0	350 (84%)
1	48 (11%)
≥2	21 (5.0%)
Miscarriage	
0	322 (77%)
1	68 (16%)
≥2	29 (7%)
Induced abortion	
0	380 (91%)
1	31 (7.4%)
≥2	8 (2.0%)
Fetal gender	
Male	217 (52%)
Female	202 (48%)
Smoking during pregnancy	
No	365 (87%)
Yes	54 (13%)
Gestational diabetes	142 (33.89%)
Gestational hypertension	42 (10.02%)
Preeclampsia	0 (0.0%)
Fetal growth restriction	5 (1.19%)

^1^ Frequency (%), mean (SD).

## Data Availability

Data are not publicly available due to privacy or ethical restrictions. Data will be made available on reasonable request. Further enquiries can be directed to the corresponding author.

## Supplementary Materials

The following supporting information can be downloaded at: https://www.mdpi.com/article/10.3390/diagnostics16193081/s1, Table S1: Questionary for the maternal fetal movements evaluation; Table S2: Multivariable models; Figure S1: Boxplots of computerized cardiotocography parameters stratified pregnant’ responses to the question “In the last 2 weeks, did the strength of your baby’s movements…? (Equal, Decreased, or Increased)”; Figure S2: Boxplots of computerized cardiotocography parameters stratified pregnant’ responses to the question “During the last 2 weeks, did you feel your baby having hiccups?”.

## Abbreviations

The following abbreviations are used in this manuscript:

FM	Fetal movements
CTG	Cardiotocography
cCTG	Computerized cardiotocography
AI	Artificial intelligence
LTV	Long-term variability
STV	Short-term variability
FHR	Fetal heart rate
