# Supplementary material for: Maternally Perceived Fetal Movement and Computerized Cardiotocography Parameters: What’s the Link? A Multicenter Pilot Study on Fetal Health
Source: Diagnostics (Basel). 2026 Sep 23;16(19):3081. doi: 10.3390/diagnostics16193081 (PMC13664599; doi:10.3390/diagnostics16193081)

Figure S2. Boxplots of computerized cardiotocography parameters stratified pregnant' responses to the question "During the last 2 weeks, did you feel your baby having hiccups?".

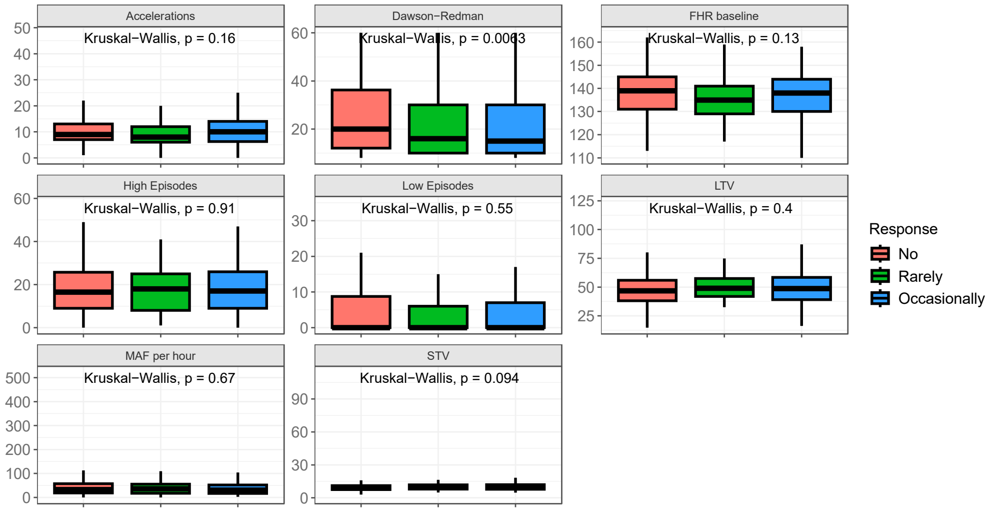

Supplement: Supplementary file 1 [file diagnostics-16-03081-s001.zip › Figure S2.pdf]
