# Supplementary material for: Maternally Perceived Fetal Movement and Computerized Cardiotocography Parameters: What’s the Link? A Multicenter Pilot Study on Fetal Health
Source: Diagnostics (Basel). 2026 Sep 23;16(19):3081. doi: 10.3390/diagnostics16193081 (PMC13664599; doi:10.3390/diagnostics16193081)
Supplement: Supplementary file 1 [file diagnostics-16-03081-s001.zip › Table S1.pdf]

Table S1. Questionary for the maternal fetal movements evaluation.

Question	Response
Was there any time from 26 weeks of pregnancy that your baby's movements were less than usual?	No<br>Once<br>Two Times<br>Three or more times
In the last 2 weeks did the strength of your baby's movements?	Increase<br>Decrease<br>Stay the same<br>Unsure
In the last 2 weeks did the frequency of your baby's movements?	Increase<br>Decrease<br>Stay the same<br>Unsure
During the last 2 weeks did you notice anytime that your baby was more vigorous than usual?	No<br>Once<br>More than once
During the last 2 weeks did you feel you baby having hiccups?	Yes<br>No<br>Unsure
