# Supplementary material for: Maternally Perceived Fetal Movement and Computerized Cardiotocography Parameters: What’s the Link? A Multicenter Pilot Study on Fetal Health
Source: Diagnostics (Basel). 2026 Sep 23;16(19):3081. doi: 10.3390/diagnostics16193081 (PMC13664599; doi:10.3390/diagnostics16193081)
Supplement: Supplementary file 1 [file diagnostics-16-03081-s001.zip › Table S2.pdf]

Table S2. Multivariable models.

Variable	Adjusted estimate	95% CI	p-value
**Model 1 - LTV, ms (linear regression, adjusted beta; n = 238; overall p for FM perception = 0.101)**			
Reduced FM since 26 weeks: one time (ref. no)	-5.69	-11.69 to 0.31	0.063
two times	-0.30	-7.65 to 7.05	0.937
three or more times	-7.47	-15.43 to 0.50	0.066
Pre-pregnancy BMI (per kg/m^2)	0.12	-0.24 to 0.48	0.512
Gestational diabetes (yes vs no)	-5.52	-11.24 to 0.21	0.059
Gestational hypertension (yes vs no)	-6.78	-16.30 to 2.74	0.162
**Model 2 - Accelerations, n (negative binomial regression, adjusted IRR; n = 417; overall p = 0.015)**			
FM frequency, last 2 weeks: increased (ref. unchanged)	1.14	1.01 to 1.29	0.040
decreased	0.90	0.76 to 1.07	0.221
Pre-pregnancy BMI (per kg/m^2)	0.99	0.98 to 1.00	0.162
Gestational diabetes (yes vs no)	1.03	0.91 to 1.16	0.677
Gestational hypertension (yes vs no)	1.11	0.92 to 1.36	0.282
**Model 3 - Time to Dawes-Redman criteria, min (Gamma regression, adjusted time ratio; n = 391; overall p = 0.025)**			
Fetal hiccup perception: occasionally (ref. not perceived)	0.85	0.74 to 0.97	0.021
often	0.85	0.73 to 0.99	0.030
Pre-pregnancy BMI (per kg/m^2)	1.00	0.99 to 1.01	0.673
Gestational diabetes (yes vs no)	0.81	0.71 to 0.91	<0.001
Gestational hypertension (yes vs no)	1.04	0.85 to 1.28	0.737

FM: fetal movements; BMI: body mass index; IRR: incidence rate ratio; CI: confidence interval. All models are adjusted for pre-pregnancy BMI, gestational diabetes, and gestational hypertension (defined by diagnosis or ongoing specific treatment). Negative binomial regression was used owing to overdispersion of the Poisson model; Gamma regression (log link) was used given the right-skewed outcome; time ratios <1 indicate a shorter time to satisfy the criteria.
